# Development of a Diagnostic Model Focusing on Esophageal Dysmotility in Patients with Systemic Sclerosis

**DOI:** 10.3390/diagnostics12123142

**Published:** 2022-12-13

**Authors:** Peiling Liu, Jing Chai, Liyi Dai, Beidi Chen, Jinxia Zhao, Ming Lu, Lin Zeng, Zhiwei Xia, Rong Mu

**Affiliations:** 1 Department of Rheumatology and Immunology, Peking University Third Hospital, No. 49 Huayuan North Road, Beijing 100191, China; 2 Department of Respiratory and Critical Care Medicine, Peking University Third Hospital, Beijing 100191, China; 3 Department of Infectious Diseases, Peking University Third Hospital, Beijing 100191, China; 4 Research Center of Clinical Epidemiology, Peking University Third Hospital, Beijing 100191, China; 5 Department of Gastroenterology, Peking University Third Hospital, Beijing 100191, China

**Keywords:** systemic sclerosis, esophageal dysmotility, esophageal dilatation on chest CT

## Abstract

Objective. Esophageal dysmotility is a common and neglected complication of systemic sclerosis (SSc) associated with poor prognosis, while the assessment remains a challenge. We aimed to develop a diagnostic model for esophageal dysmotility in SSc patients that provides individualized risk estimates. Methods. Seventy-five SSc patients who underwent high-resolution manometry (HRM) were included in the study. Esophageal widest diameter (WED) was measured on a chest CT scan. Esophageal parameters between patients with and without esophageal dysmotility were compared. Multivariate logistic regression analysis and Least Absolute Shrinkage and Selection Operator (LASSO) regression were used to fit the model. The diagnostic model was evaluated by discrimination and calibration. Internal validation was estimated using the enhanced bootstrap method with 1000 repetitions. Results. Sixty-one systemic sclerosis patients (81.3%) were diagnosed with esophageal dysmotility according to the Chicago Classification v 3.0. The diagnostic model for evaluating the probability of esophageal dysmotility integrated clinical and imaging features, including disease duration, ILD, and WED. The model displayed good discrimination with an area under the curve (AUC) of 0.923 (95% CI: 0.837–1.000), a Brier score of 0.083, and good calibration. A high AUC value of 0.911 could still be achieved in the internal validation. Conclusion. The diagnostic model, which combines the disease duration, ILD, and imaging feature (WED), is an effective and noninvasive method for predicting esophageal dysmotility in SSc patients.

## 1. Introduction

The gastrointestinal tract, especially the esophagus, is commonly affected by systemic sclerosis (SSc). Esophageal involvement, mainly manifested by gastroesophageal reflux disease and esophageal dysmotility, may occur in up to 90% of SSc patients [1,2], among which esophageal dysmotility has a prevalence between 50% and 90% in diverse groups of SSc patients [3,4]. Esophageal dysmotility may reduce the quality of life and lead to depression [3,5,6]. In particular, there is also growing evidence that microaspiration resulting from esophageal dysmotility induces interstitial lung disease and deteriorates lung function [3,4,7,8], and progressive esophageal dysmotility may negatively impact posttransplant outcomes and survival [9]. Although esophageal dysmotility is deemed an important part of the Scleroderma Clinical Trials Consortium Impairment Index to quantitate the organ damage in SSc [10], an appropriate screening strategy for esophageal dysmotility in clinical practice is still a problem.

High-resolution manometry (HRM) has replaced traditional manometry as the most accurate tool for evaluating esophageal motility, and it can also help identify the type of esophageal motility disorders. However, there are no specific recommendations on the utility of HRM for SSc in clinical practice. Although some studies suggest early HRM testing for SSc patients, there is no evidence that this practice impacts long-term outcomes. Subsequently, HRM is difficult to be widely used for screening esophageal dysmotility because it is invasive and may increase the economic burden. In this situation, the evaluation of the esophagus involvement is still mostly esophageal symptom-based, such as dysphagia, poor eating, heartburn, and nausea or vomiting [11,12]. However, prior studies report that there is a discordance regarding the correlation between manometric findings and patients’ symptoms, and up to 40% of SSc patients with esophageal dysmotility could be asymptomatic [13,14,15]. Despite these challenges, there is still a strong clinical need to detect esophageal dysmotility in SSc patients due to its insidious onset and potential hazards.

Esophageal dilatation has been considered a cardinal feature of systemic sclerosis [16,17], and esophageal diameter on chest CT could be used as an indicator for screening esophageal dysmotility. Pitrez et al. applied the diagnostic standard of infra-aortic esophagus diameter >9 mm to assess esophageal dysmotility as assessed by radionuclide scintigraphy in 76 SSc patients, with a sensitivity of 83.1% and a specificity of 94.1% [18]. Vonk et al. found that the sensitivity and specificity of esophageal dilatation, defined as a luminal coronal diameter of the esophagus >10 mm arbitrarily, in 61 SSc patients that received a diagnosis of esophageal dysmotility using a barium esophagram were 71% and 61%, respectively [19]. Karamanolis et al. reported that esophageal dilatation, defined as esophageal diameter > 9 mm, was more common in patients with esophageal dysmotility than in those with normal motility based on HRM performed in 26 SSc [20]. Although there were some well-constructed studies that had reported the correlation between esophageal dilatation and esophageal motility in SSc, there are major limitations, such as failure to apply the gold standard, i.e., HRM, for evaluation, or small sample size [18,19,20]. Besides, previous studies empirically defined esophageal dilatation, making it difficult for clinicians to determine which of those various thresholds could better be applied in clinical practice to diagnose esophageal dysmotility in patients with SSc.

In this study, we recruited an SSc cohort with HRM results to explore practical and effective indicators for esophageal dysmotility. We established a precise diagnostic model to estimate esophageal dysmotility based on the widest esophageal diameter.

## 2. Materials and Methods

### 2.1. Study Population

Seventy-five patients were recruited from the PKUTH SSc Cohort at the Department of Rheumatology and Immunology, Peking University Third Hospital (PKUTH), between January 2015 and December 2021. All patients met the 2013 ACR/EULAR systemic sclerosis classification criteria [21] and were categorized into diffuse cutaneous SSc (dcSSc) and limited cutaneous SSc (lcSSc) according to the LeRoy criteria [22]. Patients with overlap syndrome, previous upper gastrointestinal surgery, and who could not undergo HRM and chest CT for different reasons were excluded. This study was approved by the Ethics Committee of Peking University Third Hospital (M2022107) and was exempted from informed consent.

Clinical and laboratory data were collected from medical records. Disease duration was the interval between the first non-Raynaud symptom and the date of HRM. Esophageal symptoms were recorded as dysphagia, heartburn, reflux, chest pain, nausea, or vomiting [23,24]. Telangiectasia is visible macular dilated superficial blood vessels, which collapse upon pressure and slowly fill when pressure is released. Digital pitting ulcers or scars were distal to or at the proximal interphalangeal joint not thought to be due to trauma. Interstitial lung disease (ILD) was defined by HRCT as showing >20% extent on the HRCT of the chest [10]. Pulmonary arterial hypertension (PAH) was defined as a resting mean pulmonary artery pressure ≥ 25 mmHg and pulmonary capillary wedge pressure (PCWP) ≤ 15 mmHg measured by right heart catheterization or pulmonary arterial systolic pressure (PASP) ≥ 30 mmHg as determined by echocardiography [25].

Laboratory results were also recorded, including anti-centromere antibody positivity, anti-topoisomerase I (Scl-70) antibody positivity, and concentration of albumin (Alb) and hemoglobin (Hb). Hypoalbuminemia was defined as Alb levels < 40 g/L. Anemia was defined as Hb levels < 130 g/L in men and <120 g/L in women. Current or previous use of glucocorticoids, proton pump inhibitors (PPIs), and prokinetic drugs were recorded.

### 2.2. Esophageal Measurements on Chest CT Scan

**Figure 1:**
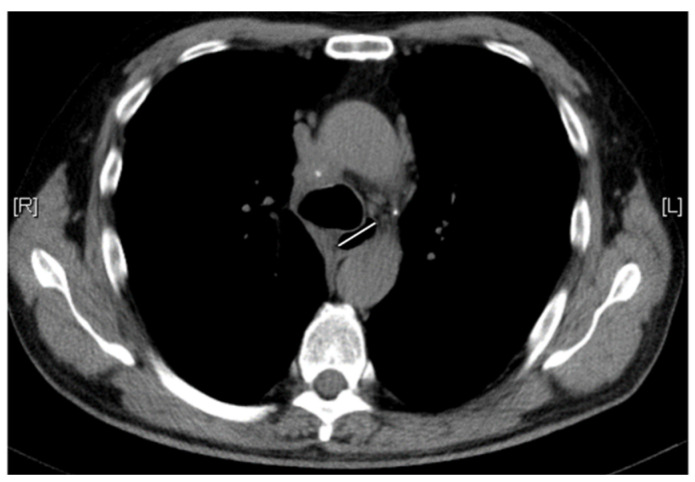
Widest esophageal diameter (WED) assessment (white line) on chest high-resolution computed tomography scans.

All patients underwent chest CT in a supine position with multidetector CT equipment (GE Discovery CT750 HD, GE Healthcare, Chicago, IL, USA). Esophageal parameters were independently measured by two radiologists blinded to clinical data when images were set as a mediastinal window (window width 350 HU, window level 50 HU) with a section thickness of 5 mm. The widest esophageal diameter (WED) is defined as the maximum distance between the esophageal mucosa on axial CT images in the supine position [26], as shown in Figure 1. The WED was measured by two rheumatologists who were blinded to clinical and laboratory data under the direction of an experienced radiologist.

### 2.3. Clinical Outcome

The primary outcome was esophageal dysmotility, diagnosed by HRM. HRM was performed using a solid-state assembly with 36 circumferential pressure sensors spaced 1 cm apart and analyzed by a gastroenterologist using Mano View v3.01 analysis software (Sierra Scientific Instruments, Los Angeles, CA, USA). Integrated relaxation pressure (IRP), distal contraction integral, and distal latency were measured, and the Chicago Classification of esophageal motility disorders v3.0 was applied to diagnose esophageal dysmotility [27]. In this study, esophageal motility disorders were classified into the following types: ineffective esophageal motility (≥50% ineffective swallows), absent contractility (normal median IRP with 100% failed peristalsis), achalasia subtype I (median IRP > 15 mmHg and 100% failed contractions), and EGJ outflow obstruction (median IRP > 15 mmHg and some maintained peristalsis) [27].

### 2.4. Statistical Analysis

Categorical variables such as gender, dcSSc, the presence of esophageal symptoms, and other clinical manifestations were expressed as numbers (percentage) and analyzed by Chi-square tests or Fisher’s exact test. Continuous variables, including age, disease duration, BMI, and WED, were subjected to the Kolmogorov–Smirnov (K–S) test to verify normal distribution and were given as the mean (S.D.) or median (interquartile range). Comparisons between the two groups (esophageal dysmotility and normal esophageal motility) were made using the Student’s t-test or the Mann–Whitney U test.

The least absolute shrinkage and selection operator (LASSO) model was used for screening optimal predictors from the candidate variables preselected on the basis of univariate analysis. The optimal model was found via cross-validation. Then, a multivariable logistic regression model incorporating those selected predictive variables was established to estimate the coefficients of each predictor. The predictive risk of esophageal dysmotility in SSc for an individual patient can be calculated using the following formula: p = e^x^/(1 + e^x^), where x equals the sum of the products of the predictors and their coefficients. The optimal critical value of the model was determined by the Youden index. The model performance was assessed by Harrell’s concordance index (C-index), the Brier score, and a calibration curve. C-index, a generalization of AUC, was used to evaluate the discriminatory ability of the model. Internal validation of the model was performed using the enhanced bootstrap method (with 1000 repetitions), and the relative corrected C-index and Brier score was calculated. Calibration curves were subsequently performed to assess the calibration of the model. We presented the final model results in the form of a web page via Shiny apps, an online platform. The methods described in this article follow the Transparent Reporting of a Multivariable Prediction Model for Individual Prognosis or Diagnosis (TRIPOD) statement [28].

All statistical analyses were performed with SPSS Statistics (v. 22) and R software (v. 3.6.3). *p* value less than 0.05 was statistically significant.

## 3. Results

### 3.1. Prevalence of Esophageal Motility Abnormalities

A total of 75 patients were enrolled in this study. The patients were predominantly female (90.6%), with a mean age of 53.0 years. According to the Chicago Classification v3.0, 81.3% (61/75) of the SSc patients were diagnosed with esophageal dysmotility. Among them, absent contractility (40.0%, 30/75) was the most frequent manifestation, followed by ineffective esophageal motility (34.6%, 26/75), EGJ outflow obstruction (4.0%, 3/75) and achalasia subtype I (2.7%, 2/75). Esophageal motility was normal in 18.7% (14/75) of the patients.

### 3.2. Comparison of Clinical Characteristics between Patients with Esophageal Dysmotility and Normal Motility

In order to further investigate the clinical implications of esophageal dysmotility in SSc, a univariate analysis was performed using the demographic and clinical characteristics of patients (Table 1). Except for disease duration [3.5 (1.0, 7.8) years vs. 0.70 (0.24, 4.0) years, *p* = 0.003)], no significant difference was found in gender, age, or BMI between the patients with and without esophageal dysmotility. Importantly, the incidence of esophageal symptoms in the patients with esophageal dysmotility was not significantly different from those with normal esophageal motility (62.3% vs. 35.7%, *p* = 0.070). Meanwhile, 37.7% (23/61) of the patients with esophageal dysmotility had no esophageal symptoms. In addition, the presence of digital ulcers and ILD in patients with esophageal dysmotility was higher than that in patients with normal esophageal motility (39.3% vs. 7.1%, *p* = 0.047; 82.0% vs. 57.1%, *p* = 0.045). There were no differences in other clinical manifestations between the two groups (Table 1). Regarding laboratory parameters and medications, neither the antibody profile nor administration of gastrointestinal medicines, such as PPIs and prokinetic drugs, differed between the two groups (Table 1).

**Figure 2:**
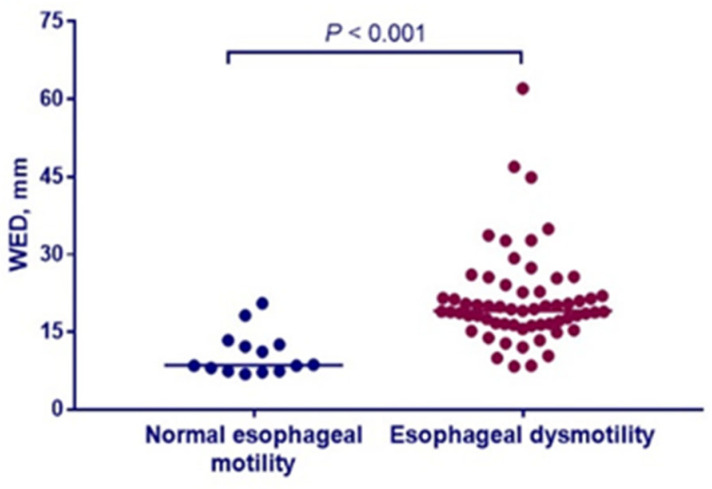
Widest esophageal diameter (WED) was elevated in SSc patients with esophageal dysmotility (n = 61) compared to those with normal esophageal motility (n = 14).

The results of analyses comparing esophageal diameter on chest CT between patients with and without esophageal dysmotility are shown in Figure 2. WED in patients with esophageal dysmotility ranged from 8.3 mm to 62.1 mm, compared with 6.9 mm to 20.5 mm in those without esophageal dysmotility. The SSc patients with esophageal dysmotility exhibited higher WED than the patients without esophageal dysmotility [19.1 (16.4, 22.7) mm vs. 8.5 (7.4, 12.8) mm, *p* < 0.001].

### 3.3. Development of Diagnostic Model

**Figure 3:**
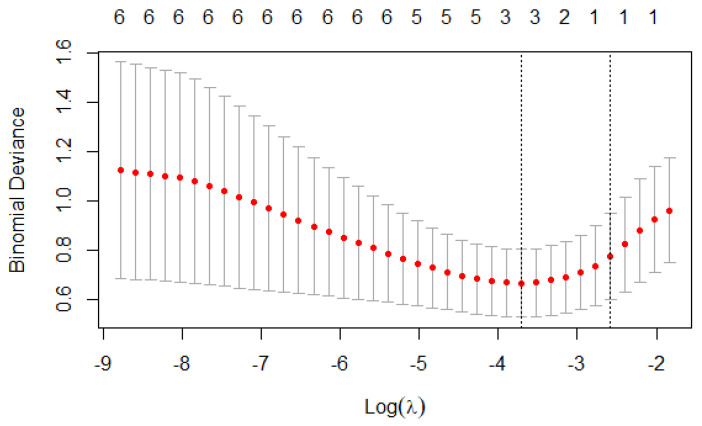
Selection of demographic and clinical features using the LASSO binary logistic regression model. Optimal parameter (λ) selection in the LASSO model using minimum criteria. The partial likelihood deviance (binomial deviance) curve was plotted versus log (λ). Dotted vertical lines were drawn at the optimal values by using the minimum criteria and 1 SE of the minimum criteria (the 1-SE criteria). LASSO: least absolute shrinkage and selection operator; SE: standard error.

According to the expert opinion and results of univariate analysis, the following variables: disease duration, digital ulcers, ILD, PAH, WED, and esophageal symptoms were selected into the LASSO regression model. When λ = 0.0295, which is the smallest estimation error of the model, was applied, there were three final variables (disease duration, ILD, and WED) in the final model. A cross-validated error plot of the LASSO regression model is shown in Figure 3. All SSc patients were used to develop the model, and the three predictors above were further analyzed in the multivariable logistic analysis (Table 2). The predicted risk of esophageal dysmotility in SSc for an individual patient can be calculated using the following formula: p = e^x^/(1 + e^x^), where x = −4.975 + 0.182 × disease duration + 0.376 × WED + 0.372 × ILD. The variable of ILD was coded as binary.

### 3.4. Model Performance and Internal Validation

**Figure 4:**
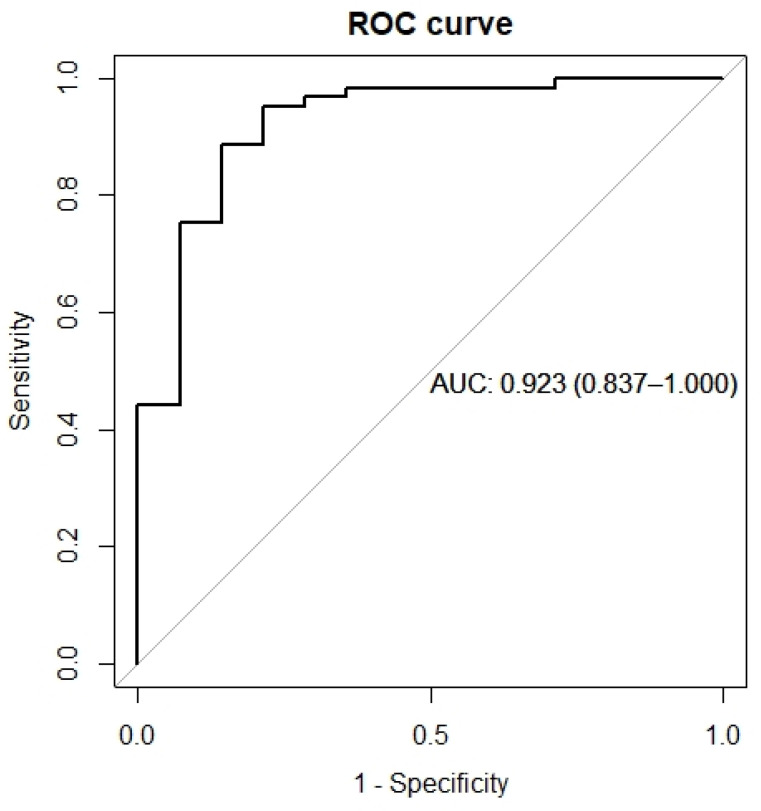
Receiver operating characteristic (ROC) curve of the diagnostic model for esophageal dysmotility in SSc.

**Figure 5:**
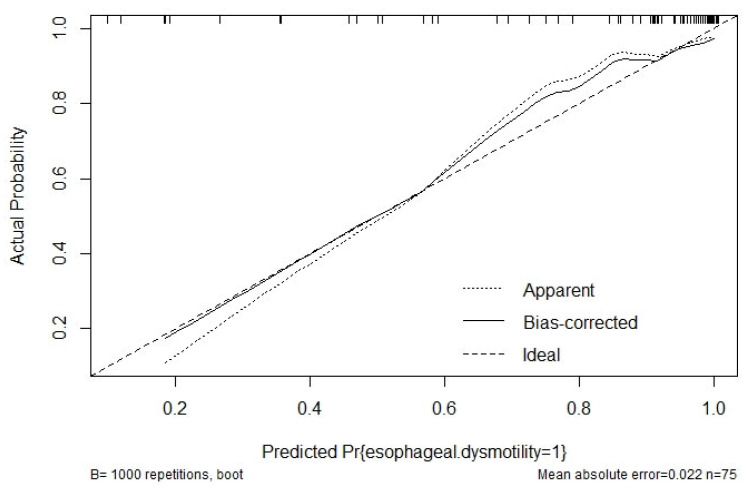
The calibration curve of the diagnostic model. The diagonal dotted line represents perfect prediction by an ideal model; the solid, dashed line represents the performance of the diagnostic model during internal validation by bootstrapping (B = 1000 repetitions).

The performance of the diagnostic model for esophageal dysmotility in SSc patients was evaluated based on discrimination and calibration. The apparent C-index for the final model was 0.923 (95% CI 0.837–1.000), and the Youden index is 0.735, as shown in (Figure 4), with a sensitivity of 88.5% and specificity of 85.7%. After bootstrapping validation, the C-index was confirmed as 0.911, which indicated that the model had good discriminatory ability. The Brier score for the final model was 0.083, and the corrected Brier score was 0.088. Calibration curves showed the comparison between the predicted risk and the observed outcome (Figure 5).

### 3.5. Visual Rendering of the Model

**Figure 6:**
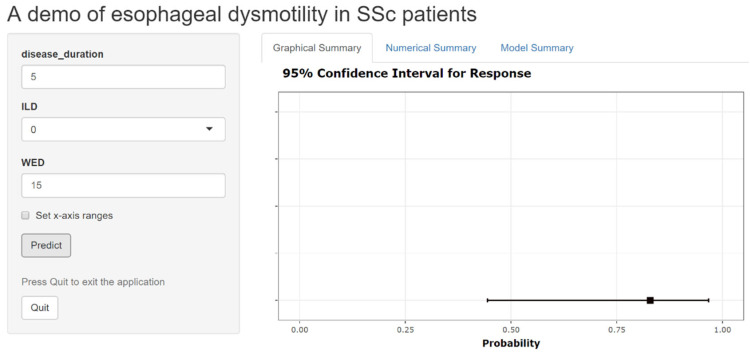
Web nomogram predicting the probability of a SSc patient with esophageal dysmotility.

Via Shiny apps, an online platform, we upload our model to the network for the convenience of other researchers to use on other equipment. The URL link is https://predictive-model-of-esophageal-dysmotility-in-ssc-patients.shinyapps.io/dynnomapp/, accessed on 1 March 2022. (Figure 6). The user can input the value of the independent variable in the input box of the independent variables, then press the’predict’ button to obtain the graphical summary and numerical summary.

## 4. Discussion

In this study, we have established a feasible clinical diagnostic model for esophageal dysmotility in SSc patients. The model incorporates routine clinical parameters (disease duration and ILD) and image indicators (WED) with relatively high predictive values. As a routine noninvasive examination, imaging data from chest CT is easily available, making this diagnostic model easy to use in clinical practice. To our knowledge, this is the first diagnostic model of esophageal dysmotility in SSc patients, which may assist clinicians with the identification of patients who probably had esophageal dysmotility, prompt recommendation of patients to perform HRM to assess the esophageal status and take timely treatment.

As one of the predictors, disease duration may be associated with diverse esophageal motor patterns in SSc. A retrospective study included a sample of 102 SSc patients and suggested that the vast majority of patients with dcSSc will have deterioration in esophageal involvement over time [29].

The presence of ILD was also included as a predictor for esophageal dysmotility. Most studies have observed a correlation between esophageal dysmotility on HRM and the presence of ILD on HRCT and DLCO < 0.8 of predicted value due to lung injury through micro-aspiration [3,4,8,14]. Meanwhile, some studies have found that esophageal dysmotility is related to the progress of pulmonary fibrosis. Panopoulos et al. conducted a follow-up study of 119 patients with early SSc patients and found that esophageal dysmotility was an independent risk factor that developed into pulmonary fibrosis within 3 and 6 years (OR was 5.6 and 4.79, respectively) [30].

Chest CT, a routine and noninvasive examination that is readily available, is a valuable screening tool for evaluating SSc-ILD. Carnevale et al. have found the change in the radiological extent of SSc-ILD was correlated to functional decline in a limited time frame [31]. HRCT is also commonly used for risk stratification and follow-up of disease severity [32]. Esophageal dilatation on chest CT is a feature of esophageal involvement in SSc [20,33]. Currently, there is still no commonly accepted definition of esophageal dilatation assessed by chest CT, and previous studies used different measurement methods and empirical cut-offs of esophageal diameter to define esophageal dilatation [17,18,26,34,35]. Accumulating evidence has suggested that SSc patients with increased WED had significant esophageal dysfunction, and increased WED was a predictive factor in ILD progression [26,34,36]. Vonk et al. considered infra-aortic esophagus coronal diameter > 9 mm as esophageal dilatation [19]. Takekoshi et al. proposed a cut-off value of 10 mm at the carinal level and 15 mm for maximum esophageal diameter [33,34]. Salaffi et al. suggested that an increased esophageal diameter (>11 mm) on chest CT is associated with SSc-ILD [34]. However, various thresholds may lead to different results [18,19], making it difficult to apply in clinical practice. Different from previous studies, we measured WED as a continuous variable and used HRM, a more accurate assessment of esophageal motility, to identify the correlation between esophageal motility and esophageal diameter.

There are several limitations of our current study. First, this is a retrospective study and lacks some useful clinical indicators such as mRSS, and gastrointestinal symptoms score questionnaires. Second, the sample size is small. Meanwhile, the results of this study were not confirmed by an external validation data set. Then, our study population was skewed towards patients with possible esophageal motility disorders, which may have biased the study results. Therefore, the generalizability of the model for the overall population of SSc patients might be limited. Future multi-center research with a larger cohort should be conducted to validate the performance of this proposed model.

## 5. Conclusions

Collectively, we have developed an effective and noninvasive diagnostic model based on three easily available clinical characteristics that predict individual risk of esophageal dysmotility in patients with SSc. Individualized estimates of risk could help clinicians identify patients with a high risk of esophageal dysmotility and prescribe appropriate treatment strategies. The model still needs to be validated and optimized with more cohorts in the future.

## Figures and Tables

**Table 1:** Demographic and clinical characteristics of SSc patients with and without esophageal dysmotility.

	Total (n = 75)	Normal Esophageal Motility (n = 14)	Esophageal Dysmotility (n = 61)	*p* Value
Age, mean (S.D.)	53.0 (13.9)	50.6 (11.7)	53.0 (14.3)	0.237
Female, n (%)	68 (90.7)	13 (92.9)	55 (90.2)	>0.999
BMI, median kg/m^2^	22.1 (19.7, 24.2)	24.8 (20.3, 26.8)	21.6 (19.5, 23.5)	0.076
Disease duration, median years,	3.5 (1.0, 7.8)	0.70 (0.24, 4.0)	4.0 (1.25, 8.0)	0.003 **
Diffuse SSc, n (%)	24 (32.0)	3 (21.4)	21 (34.4)	0.534
Raynaud’s phenomenon, n (%)	71 (94.7)	14 (100)	57 (93.4)	>0.999 ^a^
Telangiectasia, n (%)	25 (33.3)	2 (14.3)	23 (37.7)	0.173
Digital ulcers, n (%)	25 (33.3)	1 (7.1)	24 (39.3)	0.047 *
Esophageal symptoms, n (%)	43 (57.3)	5 (35.7)	38 (62.3)	0.070
ILD, n (%)	58 (77.3)	8 (57.1)	50 (82.0)	0.045 *
PAH, n (%)	14 (18.7)	0 (0)	14 (23.0)	0.059 ^a^
Prior medications				
Prednisone, n (%)	32 (42.7)	4 (28.6)	28 (45.9)	0.377
PPIs, n (%)	14 (18.7)	1 (7.1)	13 (21.3)	0.397
prokinetic drugs, n (%)	4 (5.3)	0 (0)	4 (6.6)	>0.999 ^a^
Laboratory features				
Anti-scl70, n (%)	30 (40.0)	5 (35.7)	27 (44.3)	0.560
Anti-centromere, n (%)	18 (24.0)	4 (28.6)	15 (24.6)	>0.999
Anemia, n (%)	19 (25.3)	1 (7.1)	18 (29.5)	0.163
Hypoalbuminemia, n (%)	51 (68.0)	9 (64.3)	42 (68.9)	0.741

SSc: systemic sclerosis; BMI: body mass index; ILD: interstitial lung disease; PAH: pulmonary arterial hypertension; PPIs: proton pump inhibitors; Scl-70: anti-topoisomerase I; a: *Fisher’s* exact test; *: *p* < 0.05; **: *p* < 0.01.

**Table 2:** Multivariate regression model for esophageal dysmotility in SSc.

Predictors	β Coefficient	Odds Ratio (95% CI)	*p*
Disease duration	0.182	1.199 (0.950, 1.681)	0.227
ILD	0.372	1.450 (0.159, 42.73)	0.680
WED	0.376	1.457 (1.206, 1.948)	<0.001

CI: confidential interval; ILD: interstitial lung disease; WED: widest esophageal diameter.

## Data Availability

Not applicable.
